# Factors Determining Acceptance of Internet of Things in Medical Education: Mixed Methods Study

**DOI:** 10.2196/58377

**Published:** 2025-04-10

**Authors:** Khadija Alhumaid, Kevin Ayoubi, Maha Khalifa, Said Salloum

**Affiliations:** 1 Research & Innovation Division, Rabdan Academy, Abu Dhabi, United Arab Emirates; 2 English Faculty, Higher Colleges of Technology – CERT, Al Ain, United Arab Emirates; 3 School of Computing, Skyline University College, University City, Sharjah, 1797, United Arab Emirates, 971 65441155 ext 7079

**Keywords:** collaborative learning, student, college, university, education, Internet of Things, IoT, technology acceptance model, technology optimism, TAM, experience, attitude, opinion, perception, perspective, acceptance, adoption, survey, questionnaire, ANN, deep learning, structural equation modeling, neural network, intent, use, medical education, artificial neural network, technology innovation

## Abstract

**Background:**

The global increase in the Internet of Things (IoT) adoption has sparked interest in its application within the educational sector, particularly in colleges and universities. Previous studies have often focused on individual attitudes toward IoT without considering a multiperspective approach and have overlooked the impact of IoT on the technology acceptance model outside the educational domain.

**Objective:**

This study aims to bridge the research gap by investigating the factors influencing IoT adoption in educational settings, thereby enhancing the understanding of collaborative learning through technology. It seeks to elucidate how IoT can facilitate learning processes and technology acceptance among college and university students in the United Arab Emirates.

**Methods:**

A questionnaire was distributed to students across various colleges and universities in the United Arab Emirates, garnering 463 participants. The data collected were analyzed using a hybrid approach that integrates structural equation modeling (SEM) and artificial neural network (ANN), along with importance-performance map analysis to evaluate the significance and performance of each factor affecting IoT adoption.

**Results:**

The study, involving 463 participants, identifies 2 primary levels at which factors influence the intention to adopt IoT technologies. Initial influences include technology optimism (TOP), innovation, and learning motivation, crucial for application engagement. Advanced influences stem from technology acceptance model constructs, particularly perceived ease of use (PE) and perceived usefulness (PU), which directly enhance adoption intentions. Detailed statistical analysis using partial least squares–SEM reveals significant relationships: TOP and innovativeness impact PE (β=.412, *P*=.04; β=.608, *P*=.002, respectively), and PU significantly influences TOP (β=.381, *P*=.04), innovativeness (β=.557, *P=.*003), and learning motivation (β=.752, *P*<.001). These results support our hypotheses (H1, H2, H3, H4, and H5). Further, the intention to use IoT is significantly affected by PE and usefulness (β=.619, *P*<.001; β=.598, *P*<.001, respectively). ANN modeling enhances these findings, showing superior predictive power (*R*^2^=89.7%) compared to partial least squares–SEM (*R*^2^=86.3%), indicating a more effective identification of nonlinear associations. Importance-performance map analysis corroborates these results, demonstrating the importance and performance of PU as most critical, followed by technology innovativeness and optimism, in shaping behavioral intentions to use IoT.

**Conclusions:**

This research contributes methodologically by leveraging deep ANN architecture to explore nonlinear relationships among factors influencing IoT adoption in education. The study underscores the importance of both intrinsic motivational factors and perceived technological attributes in fostering IoT adoption, offering insights for educational institutions considering IoT integration into their learning environments.

## Introduction

### Background

**Figure 1.**
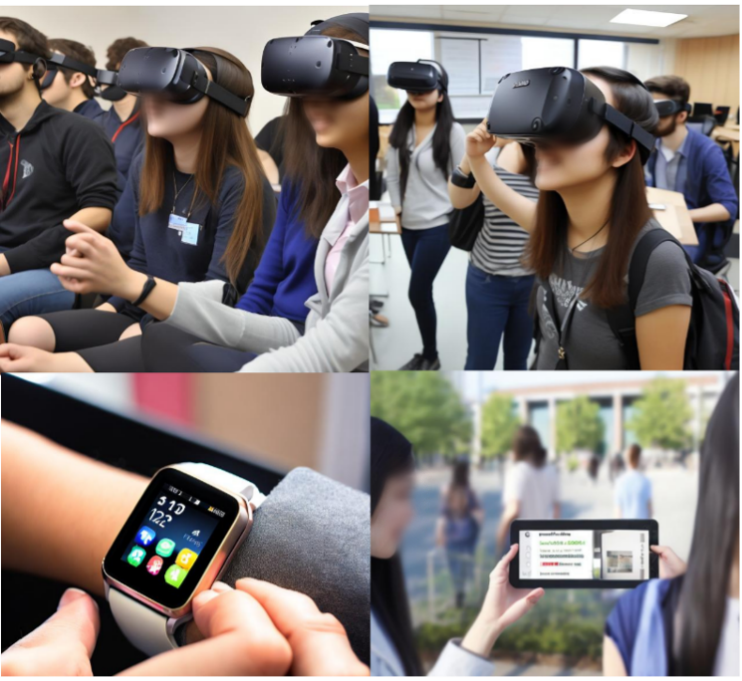
Students using augmented reality and Internet of Things technology (generated by Openart AI).

The use of Internet of Things (IoT) applications has been implemented in medical education projects [1]. IoT has the ability to completely transform the educational landscape by offering a more adaptable and quantifiable educational system that unites teachers and students under a single technological roof [2]. IoT innovation plays a significant role in transforming training at all levels, from school and college to university education [3]. Everyone, including students, instructors, and college campuses, can benefit from this innovation. Educators and administrators can leverage the power of IoT to connect people with devices and data, enabling them to gain valuable insights that have not been previously used in education. The traditional human-centered educational system has been transformed into an IoT-based one [4-6] using IoT. IoT has been leveraged to change the conventional personalized schooling system to an IoT-based system [4-6]. Figure 1 illustrates a cohort of university students actively engaging with IoT technology within an academic context. These individuals are adorned with sophisticated wearable devices, including smart glasses and smartwatches, which project information into their visual field, indicative of a digital and augmented educational milieu. The students are depicted with a palpable focus, using a portable electronic apparatus, likely for the manipulation of or interaction with the data overlay provided by their IoT devices.

The IoT has the potential to transform institutional practices and enhance learning capabilities across various levels and domains. University lecturers, students, and support staff can leverage large IoT platforms successfully. However, there is room for improvement in the utilization of IoT technology across different educational institutions. Researchers, scholars, and students can collaborate to develop IoT systems, devices, applications, and services, leading to the evolution of the educational environment as an increasingly dynamic and globally relevant subject. Better IoT deployments in colleges and universities have significantly improved positively to the creation of efficient and useful educational resources [7,8].

This research aims to explore how IoT technology is adopted and used by educators and students in universities across the Middle East. By integrating the technology acceptance model (TAM) and additional external factors, the study seeks to evaluate the effectiveness of IoT as an educational tool from the perspectives of both students and educators. Using the TAM as a metric, along with external variables, the research intends to identify and analyze the factors that influence the acceptance and use of IoT in medical education. The analysis uses a hybrid framework that combines structural equation modeling (SEM) and artificial neural networks (ANN) to examine how intrinsic motivational factors and perceived technological attributes affect IoT adoption. The SEM-ANN approach was specifically chosen to leverage the strengths of both methodologies [9,10]. SEM is highly effective in assessing the relationships between observed and latent variables, providing clarity on both the direct and indirect effects within the hypothesized model [11]. This method allows for robust statistical analysis capabilities, making it ideal for hypothesis testing and understanding the structural relationships among the theoretical constructs [12]. Conversely, ANN is used for its superior ability to model complex nonlinear relationships between variables, which are often not adequately captured by traditional linear models like SEM. ANN’s data-driven nature allows it to directly learn and adapt to these relationships from the data, thereby enhancing the model’s predictive accuracy and robustness. By integrating SEM with ANN, the study not only validates the theoretical framework through SEM’s rigorous statistical analysis but also enhances the predictive power and generalization capabilities of the model with ANN’s computational intelligence. This hybrid approach is particularly effective in exploring deeper, nonlinear interactions within the data, offering a more comprehensive understanding of the factors influencing IoT adoption. Given the complexities of modern datasets, which often exhibit nonlinear and digital behaviors among variables, this methodological integration is well-suited to achieving the research objectives and provides a justified, robust approach for the study.

The proposed hypotheses are: (1) intrinsic motivational factors like technology optimism (TOP), innovation, and learning motivation (LMT) significantly impact students’ intention to adopt IoT; (2) perceived ease of use (PE) and perceived usefulness (PU), fundamental components of the TAM, strongly predict IoT adoption intentions among students in the United Arab Emirates.

This paper is organized as follows: “Literature Review” section delves into the existing research related to the adoption of IoT technologies, highlighting key theories and previous findings that set the groundwork for this study. Section “Methodology” details the research design, sampling methods, data collection procedures, and analytical techniques used to investigate the hypotheses. Section “Findings” presents the results of the data analysis, offering quantitative insights into the factors influencing IoT adoption. Section “Discussion” interprets the findings in the context of the existing literature, discussing the implications for theory and practice. Finally, the section “Conclusions” summarizes the study’s main contributions, outlines its limitations, and suggests directions for future research. This structure is designed to provide a clear and logical progression through the topics covered, facilitating a comprehensive understanding of the study’s scope and conclusions.

### Related Work

The review of existing literature on the topic of IoT has explored both practical and theoretical aspects, suggesting a correlation between IoT and other factors such as self-efficacy, technology utilization, motivation, security, privacy, training, and more [13-19], indicating a relationship between the IoT and other elements including self-reliance, technological use, inspiration, safety, privacy, schooling, and beyond [13-19]. Likewise, research has examined the impact of IoT in conjunction with TAM and other external factors, and IoT with the help of TAM and external factors [20,21].

As IoT applications become more intricate, they can significantly impact learning. The difficulty is the IoT technology’s quick development, which requires diverse skills ranging from developing IoT applications to incorporating devices into management systems that analyze device-generated data [14,22]. Previous research has highlighted the significance of IoT in addressing challenges students encounter when using modern IoT apps and gadgets. Potential solutions include focusing on computational thinking education, assisting students in solving challenges, and providing clear instructions and training to facilitate the integration of new students with IoT devices and encourage training in it.

A different approach was taken in another study, where a workshop was offered to address the importance of IoT. The results showed that students found the workshop highly satisfactory for learning about IoT, improving problem-solving skills, and enhancing problem-solving capabilities, while also finding it enjoyable [13-15].

Several outside variables, including drive, contentment, ease of use, effectiveness, involvement, and interest, have been investigated to explore the relationship between IoT and students’ attitudes. The most significant factors are motivation and enjoyment regarding IoT technology, which are essential for its acceptance among students. While satisfaction and performance indirectly affect the application of IoT, greater contentment levels are not proportional to higher interactions [18,19]. Recent studies have used qualitative and quantitative methods to examine the uptake of IoT and have identified favorable attitudes, ease of use, contentment, affordability, basic knowledge, security, and privacy as crucial factors that influence its adoption. Apart from motivation and enjoyment, training and experience are also crucial in uptaking IoT devices, and educational training workshops can significantly improve the learning curve, digital learning, real-life applications, and problem-solving skills [16,17,23].

Previous research has explored the various benefits of implementing IoT technologies in educational settings, including the ability for teachers to gain insight into students’ performance and knowledge levels, as well as the potential for improved teaching quality. As a result, the use of IoT has the potential to greatly impact learning environments, leading to a more advanced educational landscape. This could also lead to changes in how we interact and collaborate as a society, as IoT technology continues to connect us in new ways [24,25]. While previous studies have investigated students’ attitudes toward IoT from different angles [14,22,26], there has been limited research on the importance of IoT technology from the perspective of teachers’ knowledge of the significance of IoT innovation from the educational viewpoint of instructors and how it impacts pedagogy in the classroom. Therefore, this study aims to address this gap by developing a model that combines both students’ and teachers’ attitudes to examine the effectiveness and efficiency of IoT in educational environments. By creating a framework that incorporates the viewpoints of learners and educators, this research intends to close this imbalance by analyzing the usefulness and efficacy of IoT in classrooms.

### Developing Hypotheses and Theoretical Framework

**Figure 2.**
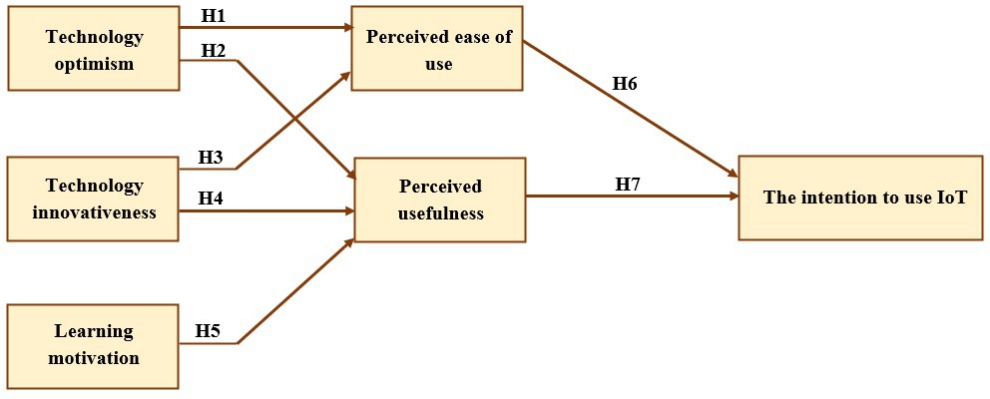
Research model. IoT: Internet of Things.

The study paradigm shown in Figure 2 describes how the inspiration for learning, technological exuberance, and technological advancements influence learners’ perceptions of the PU and ease of use (PE) of IoT devices. Such concepts have not yet been looked into in relation to IoT devices and applicability. Although earlier research has examined how these factors may affect IoT adoption plans [27], their individual effects on students’ attitudes toward this technology have not been analyzed before.

LMT is a crucial factor impacting students’ behavior in conventional and digital educational settings. It is an outside variable of the suggested approach and can be influenced by multiple factors, including the learning environment, expectations, and social values. Research has shown that LMT significantly impacts students’ academic achievements [28]. Students must therefore be driven to acquire knowledge in order to achieve the suggested intended goals [29,30]. Highly motivated learners can achieve their learning goals spontaneously and willingly. Social cognitivism emphasizes the significance of requirements, conducts, and ideologies in education.

Researchers are investigating the influence of TOP and innovation on students’ adoption of IoT in the education sector. The study aims to understand students’ attitudes and behavior toward technology by using 3 theoretical concepts: inventiveness, technological exuberance, and learning incentive. The researchers have combined the (TAM) constructs with the aforementioned variables to accomplish their study’s objectives. Several researchers have used the TAM model to evaluate the effectiveness of IoT technology and its applications [31,32]. The model explains why people accept technology based on their attitudes and beliefs and how these beliefs impact their behavioral intention toward technology use [33,34]. It is an established concept that supports embracing technology in various settings and strongly links it to technological progress [35-37]. The TAM model can forecast variables like technology elation and inventiveness that influence how well a certain technology is received [38,39]. TOP refers to users’ favorable perception of technology, while technology innovativeness (TIN) refers to users’ willingness to adopt technology early and lead to its use.

The PE and PU constructs of TAM are closely related to TOP and TIN. In the academic setting, students’ perceptions of technology can be influenced by their peers and instructors. If the student’s immediate academic circle has a positive view of a particular technology, the student will likely also develop a favorable opinion. Similarly, tech-savvy students tend to have a positive self-perception. Early on in the acceptance of technology, students are usually eager to pioneer using advanced technologies [40-44].

Furthermore, a student’s optimism about technology is linked to their level of involvement in guiding its use. Similarly, technological optimism can significantly impact a student’s attitude. IoT acceptance is greater for learners who are enthusiastic about experimenting with novel innovations. Technological innovators infrequently perceive new innovations as being challenging or outside their comprehension. Individuals are more inclined to regret not having the freedom to play around with novel technologies [45]. As a result, a number of hypotheses are put forth:

- H1: TOP affects positively the PE.
- H2: TOP affects positively the PU.
- H3: TIN affects positively the PE.
- H4: TIN affects positively the PU.
- H5: LMT affects positively the PU.
- H6: PE affects positively the intention to use Internet of Things (INT).
- H7: PU affects positively the INT.

By evaluating the association between TAM and its connected factors, this research seeks to add to a collection of current work. It has been said that it is important to measure these variables and analyze their relationship to TAM to assess the efficiency of IoT in classrooms. Past research and literature have mostly focused on preservice teachers, and there is a need to conduct more studies involving in-service teachers to enhance the practicality of the TAM model [46,47]. Thus, this study investigates the correlation between teachers’ levels of TAM and their attitudes toward IoT acceptance when working with teachers from different fields.

## Methods

### Data Collection

The data collection for this study was carried out between January 20 and March 20, 2023, throughout the academic year 2023‐2024’s winter semester, at educational institutions in the United Arab Emirates. The research team used web-based surveys to collect data, with 500 questionnaires randomly distributed. Of these, 463 surveys were answered, resulting in a response rate of 93%. Some questionnaires were rejected due to missing values. Since a few surveys had no responses, they were discarded, leaving 769 usable questionnaires, which is considered an appropriate acceptable sample size according to Krejcie and Morgan [48]. Although the sample size exceeded the minimum requirements, the research team used SEM SmartPLS (version 3.2.7; SmartPLS GmbH) and SPSS Statistics (version 23; IBM Corp) to evaluate the hypotheses and confirm the relationship between variables. It is important to note that the hypotheses were based on previous theories related to IoT, which formed the foundation of this study.

### Ethical Considerations

Ethical considerations were meticulously adhered to throughout the research process. All procedures involving human participants were approved by the institutional review board of the host universities in the United Arab Emirates (#RAREC00065), ensuring compliance with ethical standards. Informed consent was obtained from all participants before data collection, and they were informed of their rights to withdraw from the study at any time. Privacy and confidentiality were strictly maintained, with all data being anonymized and securely stored to prevent unauthorized access. Participants were not compensated for their participation, as the study involved minimal risk and was conducted as part of educational activities within the institutions involved.

### Student’s Personal Information

The demographic data of the respondents (N=463) are presented in Table 1. The data showed that 70% (n=325) of participants were female and 30% (n=138) were male. In terms of age, 42% (n=193) of participants were between 18 and 29 years, 35% (n=163) were between 30 and 39 years, 21% (n=99) were between 40 and 49 years, and 2% (n=8) were between 50 and 59 years. Regarding education, 80% (n=372) of participants held a bachelor's degree, 14% (n=62) held a master's degree, and 6% (n=29) held a doctoral degree. To obtain participants' willingness to participate, the research team used a purposive sampling approach. The participants came from various universities, academic levels, and programs relevant to this research. SPSS Statistics was used to analyze the demographic data.

**Table 1.** Demographic data of the respondents (N=463).

Category		Value, n (%)
Sex		
Female		325 (70)
Male		138 (30)
Age (years)		
Between 18 and 29		193 (42)
Between 30 and 39		163 (35)
Between 40 and 49		99 (21)
Between 50 and 59		8 (2)
Educational qualification		
Bachelor’s degree		372 (80)
Master’s degree		62 (14)
Doctorate		29 (6)

### Study Instrument

This research proposed a survey instrument for validating the hypotheses. To assess the 6 constructs of the questionnaire, an additional 18 questions were included in the survey. The origins and histories of these constructs are presented in Table 2. To make the research more relevant, the researchers modified the questions from previous studies.

**Table 2.** Measurement items.

Constructs	Items	Definition	Instrument	Sources
TIN^a^	TIN1 TIN2 TIN3	The term “technology innovativeness” describes a user’s perception that they are at the forefront of technology use. Users who are pioneers in adopting new technologies typically do not view them as complicated or difficult to comprehend. Such users may feel a sense of regret if they miss the chance to experiment with new technologies.	I accept IoT^b^ technology to be used in my daily classes. I am the only one. There is only me prepared to use IoT technology among my fellow students. I am ready to use. I am prepared to use and experiment with the latest information technologies.	[45]
TOP^c^	TOP1 TOP2 TOP3	A person’s readiness to use technology is known as technological optimism.	I am ready to test. Prepared to take the test IoT technology. To complete my assignments to finish my homework, I will be using IoT. I will learn more with the help of my preparedness to use IoT.	[2,49]
LMT^d^	LMT1 LMT2 LMT3	The concept of learning motivation is used to measure the behavioral intention to use technology. Motivation learning is composed of 4 key components, which are attention, relevance, confidence, and satisfaction. These components have been identified in previous studies [50,51].	I can improve my focus by using IoT for my daily classes. Using IoT makes me feel more confident. Using IoT for study purposes satisfies me.	[50,51]
PE^e^	PE1 PE2 PE3	The TAM^f^ was introduced by Davis [52] as a means of assessing the effectiveness and acceptance of technology. The model includes the concept of PE, which refers to the user’s perception of how effortless it is to use the technology.	IoT technology being simple will polish my skills. I can improve my learning achievements by using IoT technology. IoT is simple and easy to use.	[52]
PU^g^	PU1 PU2 PU3	Usefulness refers to PU that the users of technology may see.	IoT technology will hugely benefit me. IoT will make my abilities and skills better. Using IoT for my daily classes is beneficial.	[52]
INT^h^	INT1 INT2 INT3	An individual’s view of what others think about a certain behavior is known as a behavioral intention to use.	IoT will be my go-to for daily tasks. In the future, I will be using IoT. IoT technology will be my recommendation to every student.	[53]

a TIN: technology innovativeness.

b IoT: Internet of Things.

c TOP: technology optimism.

d LMT: learning motivation.

e PE: perceived ease of use.

f TAM: technology acceptance model.

g PU: perceived usefulness.

h INT: intention to use Internet of Things.

### Survey Structure

The questionnaire survey given to the students has 3 sections. Three components make up the survey that is provided to learners: (1) personal data is the focus of the first section, (2) the general question related to the “Intention to Use IoT” is the second section, and (3) 15 items that deal with TIN, TOP, LMT, PE, and PU is present in the third section.

A 5-point Likert scale was used to assess the 18 items, with response options ranging from 1=strongly disagree to 2=disagree, 3=neutral, 4=agree, and 5=strongly agree.

## Results

### Data Analysis

In this study, the gathered data were analyzed using SmartPLS (version 3.2.7) software through the partial least squares–structural equation modeling (PLS-SEM) technique [54-56]. The evaluation consisted of 2 stages: the measurement model and the structural model [57,58]. PLS-SEM was selected for this research after considering several factors.

The selection of PLS-SEM for this study was based on several reasons. First, PLS-SEM is preferred when the study aims to build on an existing theory [59]. Second, PLS-SEM is effective in handling complex models in exploratory research. Third, PLS-SEM analyzes the entire model as a single entity rather than dividing it into components [60]. Finally, PLS-SEM allows for the simultaneous analysis of structural and measurement models, leading to more accurate results [61].

### Convergent Validity

Hair et al [57] suggested that to assess the measurement model, it is important to examine the construct reliability (including Cronbach ɑ and composite reliability) and validity (including convergent and discriminant validity). Table 3 shows that the Cronbach ɑ readings are greater than the suggested criterion of 0.7, and vary from 0.797 to 0.858 [62], indicating good construct reliability. Similarly, the proposed criterion is also greater than the composite reliability numbers, which vary from 0.735 to 0.858 [63]. Factor loading and average-variance extracted (AVE) analysis are required to evaluate convergent validity [57]. The factor loading values in Table 3 are higher than the recommended threshold of 0.7, and the AVE readings are greater than the suggested limit of 0.5, spanning from 0.556 to 0.712. These results suggest that there is convergent validity.

**Table 3.** Convergent validity results which assures acceptable values (factor loading, CA^a^, CR^b^, Dijkstra-Henseler’s ρ ≥0.70, and AVE^c^ >0.5).

Constructs and items		Factor loading	CA	CR	AVE
TIN^d^			0.856	0.824	0.608
TIN1		0.723			
TIN2		0.873			
TIN3		0.858			
TOP^e^			0.842	0.858	0.701
TOP1		0.804			
TOP2		0.816			
TOP3		0.801			
LMT^f^			0.815	0.851	0.712
LMT1		0.765			
LMT2		0.844			
LMT3		0.758			
PE^g^			0.797	0.735	0.662
PE1		0.858			
PE2		0.825			
PE3		0.758			
PU^h^			0.858	0.853	0.556
PU1		0.801			
PU2		0.829			
PU3		0.732			
INT^i^			0.825	0.843	0.612
INT1		0.812			
INT2		0.721			
INT3		0.749			

a CA: Cronbach ɑ.

b CR: composite reliability.

c AVE: average-variance extracted.

d TIN: technology innovativeness.

e TOP: technology optimism.

f LMT: learning motivation.

g PE: perceived ease of use.

h PU: perceived usefulness.

i INT: intention to use Internet of Things.

### Discriminant Validity

To assess discriminant validity, 2 criteria were recommended: the Heterotrait-Monotrait ratio and the Fornell-Larcker criterion [57]. According to Table 4, the Fornell-Larcker criterion is met, as the AVE and its square root for each construct exceed its correlation with other constructs [64]. Table 5 displays the Heterotrait-Monotrait ratio results, which demonstrate that each construct’s value is below the threshold value of 0.85 [65]. This suggests that discriminant validity exists, and the measurement model’s reliability and validity were confirmed without any issues. As a result, the collected data can be used for analyzing the structural model.

**Table 4.** Fornell-Larcker scale.

	TIN^a^	TOP^b^	LMT^c^	PE^d^	PU^e^	INT^f^
TIN	0.864^g^	—^h^	—	—	—	—
TOP	0.675	0.883^g^	—	—	—	—
LMT	0.182	0.263	0.723^g^	—	—	—
PE	0.664	0.245	0.236	0.861^g^	—	—
PU	0.664	0.283	0.373	0.313	0.812^g^	—
INT	0.540	0.573	0.275	0.407	0.286	0.890^g^

a TIN: technology innovativeness.

b TOP: technology optimism.

c LMT: learning motivation.

d PE: perceived ease of use.

e PU: perceived usefulness.

f INT: intention to use Internet of Things.

g These values represent the square root of the average variance extracted for each construct, according to the Fornell-Larcker criterion. They are placed diagonally to demonstrate discriminant validity. A construct should share more variance with its indicators than with other constructs (off-diagonal correlations).

h Not applicable.

**Table 5.** Heterotrait-Monotrait ratio.

	TIN^a^	TOP^b^	LMT^c^	PE^d^	PU^e^	INT^f^
TIN	—^g^	0.355	0.473	0.113	0.741	0.336
TOP	0.355	—	0.406	0.512	0.579	0.512
LMT	0.473	0.406	—	0.702	0.559	0.021
PE	0.113	0.512	0.702	—	0.328	0.363
PU	0.741	0.579	0.559	0.328	—	0.486
INT	0.336	0.512	0.021	0.363	0.486	—

a TIN: technology innovativeness.

b TOP: technology optimism.

c LMT: learning motivation.

d PE: perceived ease of use.

e PU: perceived usefulness.

f INT: intention to use Internet of Things.

g Not applicable.

### Hypotheses Testing Using PLS-SEM

**Figure 3.**
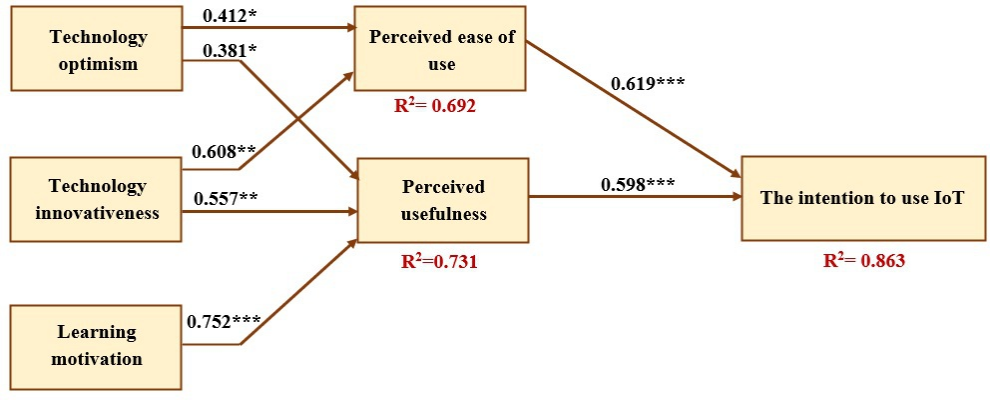
The path coefficients of the research model. IoT: Internet of Things. *P<.05; **P<.01; ***P<.001.

To assess whether the theoretical constructs of the structural model are interconnected, the study used Smart PLS with maximum likelihood estimation to create a structural equation model [66]. The proposed hypotheses were then analyzed using this model. The results indicated a high level of predictive power for the model, with 86.3% of the variance in INT being accounted for, as shown in Figure 3 and Table 6.

Table 7 provides information on the β values, *t* values, and *P *values of all developed hypotheses based on the findings produced using the PLS-SEM technique. The researchers have confirmed each hypothesis. The empirical data supported H1, H2, H3, H4, H5, H6, and H7 following the data analysis hypotheses. This study demonstrates that TOP and TIN have a noteworthy influence on PE with respective regression coefficients (β=.412, *P*=.04), and (β=.608, *P*=.002), supporting H1 and H3. Moreover, the results indicate that PU is significantly impacted by TOP (β=.381, *P*=.04), TIN (β=.557, *P*=.003), and LMT (β=.752, *P*<.001), supporting hypotheses H2, H4, and H5, respectively. Finally, the study reveals that the relationship between PE and PU significantly affects INT with respective regression coefficients (β=.619, *P*<.001) and (β=.598, *P*<.001), supporting H6 and H7.

**Table 6.** The *R*^2^ values for the endogenous latent variables.

Construct	*R* ^2^	Results
PE^a^	0.692	High
PU^b^	0.731	High
INT^c^	0.863	High

a PE: perceived ease of use.

b PU: perceived usefulness.

c INT: intention to use Internet of Things.

**Table 7.** Results of hypotheses testing for the research model at significance levels of .01 and .05.

H	Relationship	Path	*t* test (*df*)	*P* value	Direction	Decision
H1	TOP^a^ -> PE^b^	0.412	5.552 (461)	.048	Positive	Supported
H2	TOP -> PU^c^	0.381	4.843 (461)	.04	Positive	Supported
H3	TIN^d^ -> PE	0.608	10.247 (461)	.002	Positive	Supported
H4	TIN -> PU	0.557	9.358 (461)	.003	Positive	Supported
H5	LMT^e^ -> PU	0.752	14.450 (461)	<.001	Positive	Supported
H6	PE -> INT^f^	0.619	16.753 (461)	<.001	Positive	Supported
H7	PU -> INT	0.598	14.195 (461)	<.001	Positive	Supported

a TOP: technology optimism.

b PE: perceived ease of use.

c PU: perceived usefulness.

d TIN: technology innovativeness.

e LMT: learning motivation.

f INT: intention to use Internet of Things.

### ANN Results

**Figure 4.**
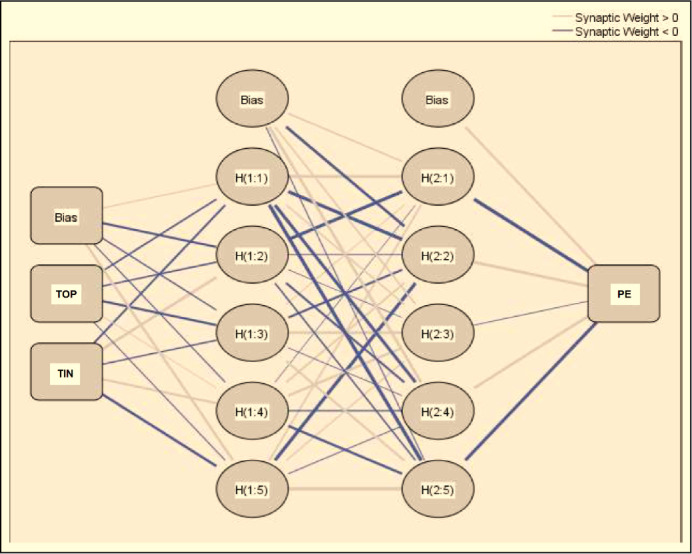
ANN model for predicting PE. ANN: artificial neural network; PE: perceived ease of use; TIN: technology innovativeness; TOP: technology optimism.

The predictors identified during the PLS-SEM analysis are further investigated through ANN analysis using SPSS software. The PLS-SEM analysis identified TIN, TOP, LMT, PE, and PU as critical factors; therefore, ANN analysis also considers these 3 factors only. The structure of the ANN model is based on behavioral intention as an output neuron and TIN, TOP, LMT, PE, and PU as input neurons (Figures4^6^). ANN model supported deep learning in all the output neuron modes through its 2-hidden layer deep structure [67,68]. The researcher applied the activation function of the sigmoid function to hidden neurons, as well as output neurons, keeping the values of input and output neurons between [0, 1]; this allowed the researcher to obtain better performance from the research model [69,70]. He also applied the 10-fold cross-validation method to training and testing data in the ratio of 80:20 to ensure that there is no overfitting in the ANN model [71]. The researcher evaluated the root mean square of error (RMSE) to test the neural network model for accuracy. The training data showed an RMSE value of 0.1388 for the ANN model while the testing data showed an RMSE value of 0.1439. The training and testing data showed only slight variance in the RMSE values and SD values (ie, 0.0043 and 0.0096). Hence, we can infer that using the ANN model enhances the accuracy of the research model.

**Figure 5.**
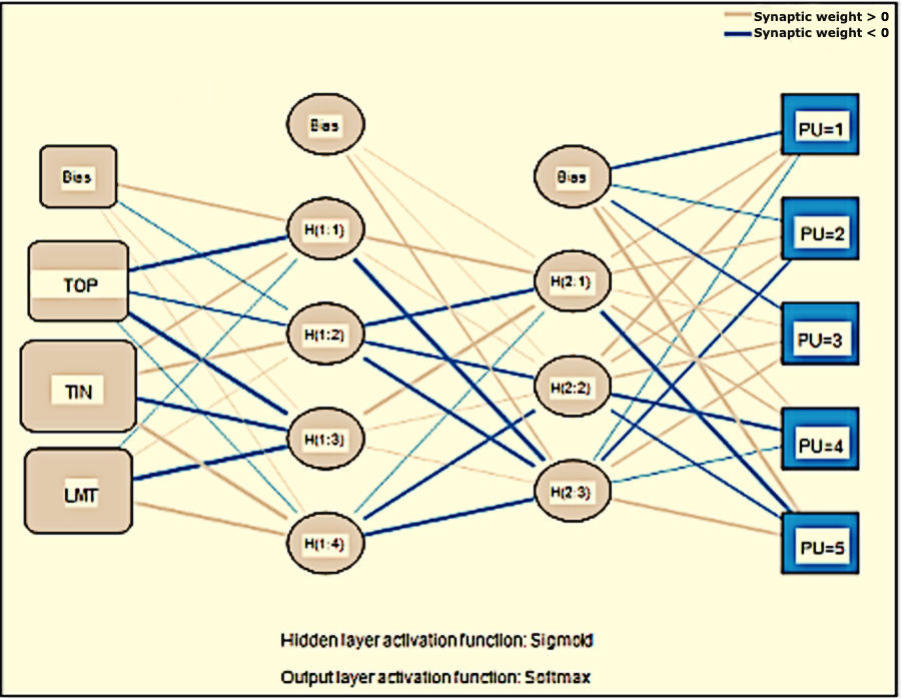
ANN model for predicting PU. ANN: artificial neural network; LMT: learning motivation; PU: perceived usefulness; TIN: technology innovativeness; TOP: technology optimism.

**Figure 6.**
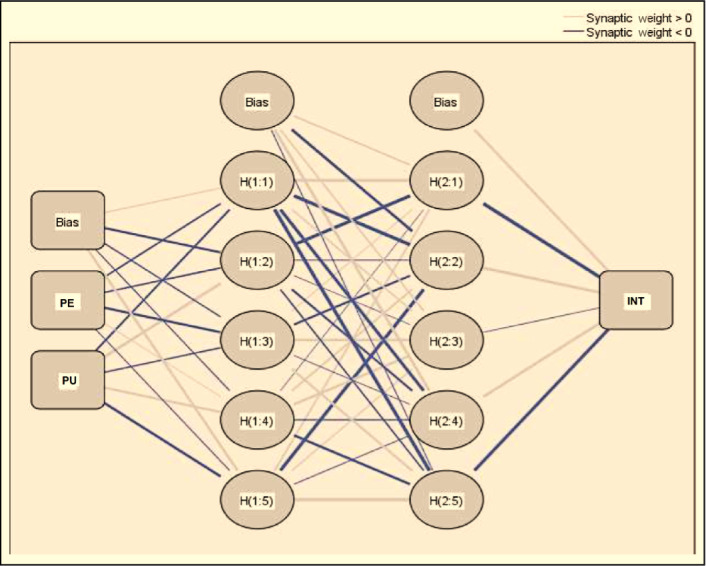
ANN model for predicting INT. ANN: artificial neural network; PE: perceived ease of use; PU: perceived usefulness; INT: intention to use Internet of Things.

### Sensitivity Analysis

The researcher estimated the value of normalized importance by comparing each predictor’s average value with the maximum mean value depicted among all predictors stated in percentage form. The values of normalized importance and mean importance computed for the predictors involved in ANN modeling are recorded in Table 8. This table also depicts the outcomes of the sensitivity analysis which identifies the predictor of PU to have the most significant impact on behavioral intention; the second most significant impact was imposed by TOP while the least impact was imposed by PE. ANN application was additionally assessed for its accuracy and performance by computing the goodness-of-fit for authentication and validation of the application. The ANN application uses goodness-of-fit just as PLS-SEM analysis uses *R*^2^ [72,73]. The predictive powers of both the applications are compared where ANN analysis outperforms the other one with a predictive power of (*R*^2^=89.7%) against the PLS-SEM predictive power of (*R*^2^=86.3%). Hence, ANN analysis can explain endogenous constructs more effectively than PLS-SEM. Moreover, ANN analysis is based on deep learning and has the potential to better identify nonlinear associations among constructs which results in variances in the predictive powers of the ANN and PLS-SEM methods.

**Table 8.** Independent variable importance.

	Importance	Normalized importance (%)
TIN^a^	0.335	83.1
TOP^b^	0.463	94.8
LMT^c^	0.361	77.5
PE^d^	0.118	19.2
PU^e^	0.539	100

a TIN: technology innovativeness.

b TOP: technology optimism.

c LMT: learning motivation.

d PE: perceived ease of use.

e PU: perceived usefulness.

### Importance-Performance Map Analysis

**Figure 7.**
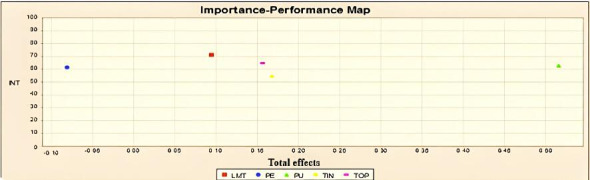
Importance-performance map analysis results. INT: intention to use Internet of Things; LMT: learning motivation; PE: perceived ease of use; PU: perceived usefulness; TIN: technology innovativeness; TOP: technology optimism.

This study has used importance-performance map analysis (IPMA) as an advanced approach in PLS-SEM, which used behavioral intention as the main variable. As suggested by Ringle and Sarstedt [74], a better interpretation of the results of PLS-SEM is possible by using IPMA. A substitute way to only test the path coefficients (ie, importance measure), the average value of the latent constructs, and their indicators (ie, performance measure) are also included in the IPMA [74]. According to IPMA, the total effects reflect the predecessor factors’ importance in developing the target factor (ie, behavioral intention), while the average of latent constructs’ values is a reflection of their performance. The IPMA findings are reported in Figure 7. The estimation of the importance and performance of the 5 factors (ie, TIN, TOP, LMT, PE, and PU) has been shown in this table. According to the findings, the PU has been reported to have the largest values in terms of both importance and performance measures. Furthermore, it can be clearly seen that TIN has the second largest values in terms of both importance and performance measures. The third largest value was reported in the case of TOP in terms of the importance measure; however, it has the smallest value on the performance measure. Relatively, the opposite scenario was reported in the case of PE, as it had the lowest value on the importance measure.

## Discussion

### Principal Findings

The main findings of this study indicate that the intention to adopt IoT technologies is positively influenced by both intrinsic motivational factors and TAM constructs. Specifically, TOP, innovation, and LMT play crucial roles at the initial level of influencing IoT adoption. At a secondary level, the perceived ease of use and PU, core elements of the TAM, directly enhance the INT technologies. Further analysis using ANNs and IPMA highlighted PU as a particularly significant predictor of IoT use intentions.

Based on these results, it is evident that technology features are critical in shaping users’ PE and PU toward IoT applications and tools. The positive influence of TOP, innovation, and LMT is reflected across all model variables, suggesting that well-designed technology features can significantly boost IoT acceptance. The TAM framework effectively provides a user-friendly experience that meets users’ value expectations and fosters positive emotional responses, which in turn, positively impacts their INT. Additionally, the significant positive impact of technology features on TOP boosts users’ trust in the system’s quality, further influencing their willingness to engage with IoT systems. The social aspect of technology use, where familiarity influences adoption, also plays a pivotal role in the INT features. Technology innovation contributes significantly to perceived ease and usefulness, enhancing trust and satisfaction during the user experience, thus fostering a conducive environment for IoT adoption.

The confirmation of hypotheses H1-H5 supports the proposed conceptual model and the proposed hypotheses [75-77]. Previous research has also produced results that are consistent with these findings. However, other studies have shown that the lack of sufficient security and privacy are major challenges that may hinder the deployment of IoT in education [63,64], and to reduce these obstacles, future efforts to implement IoT in education must consider these factors. Although IoT has not been widely adopted in resource-limited countries, scholars need to examine the factors affecting its adoption to enable effective deployment. Therefore, scholars need to investigate the use and adoption of these technologies in other domains.

### Managerial Implications

The adoption of IoT in educational settings demands that administrators and educational leaders ensure their faculty is proficient in using IoT technologies effectively in the classroom. This includes competencies in handling relevant technology tools, understanding pedagogical integration, and applying these technologies within various teaching scenarios. To support this, institutions should offer targeted professional development that focuses on both the technical and educational aspects of IoT. Additionally, management should consider the infrastructure upgrades necessary to support IoT technologies, such as improved wireless networks and enhanced security measures to protect student data [78,79].

### Practical Implications

The practical applications of this study highlight the necessity for educational curricula to evolve alongside technological advancements. Institutions offering programs in computer science and engineering should integrate IoT courses to prepare students for the demands of the workforce, which increasingly relies on IoT technologies. Moreover, schools should align their IoT strategies with real-world applications, providing students with hands-on opportunities to work with IoT in context. This could include partnerships with IoT companies or practical projects that allow students to solve real problems using IoT solutions [80,81].

### Theoretical Implications

This study contributes to the academic understanding of technology adoption by confirming the significant role of intrinsic motivational factors and TAM constructs in the adoption of IoT technologies. By highlighting the dual influence of personal motivation and perceived technological attributes, this research extends existing models of technology acceptance. Furthermore, the use of advanced analytical methods such as ANN and IPMA provides a deeper insight into the nonlinear relationships among the constructs, offering a nuanced perspective that can inform future research in technology adoption theories. This could encourage scholars to explore how different educational contexts or cultural backgrounds influence the adoption and effective use of emerging technologies like IoT [82,83].

### Conclusions

IoT technology has fundamentally altered the tech and business industries, laying the groundwork for the creation of intelligent societies and advancing social and economic development. The IoT has advanced quickly and significantly. The research team developed the PE and PU frameworks for Arab customers using path estimation and modeling of structural equations depending on their responses to analyze individuals’ acceptance of IoT. In addition to additional factors including technology exuberance, advancements in technology, and academic drive, the research additionally looked at the effects of TAM components on these factors. These factors and TAM components were found to be directly related to the study. According to the research, the incentive to learn, technological advancement, and technological positiveness all had a substantial influence on PE and PU. While earlier research has demonstrated that TAM components affect the motivation to use IoT, this research has investigated how other external factors, including social factors like LMT, can affect these constructs. The study used a conceptual model to examine users’ attitudes toward IoT adoption, with 2 levels of analysis. The first level focused on social attitudes, particularly the impact of the incentive to learn as a standalone factor. The second stage looked at how personal traits influenced technological positivism and inventiveness, influencing customer needs and IoT interactions. Subsequent research could look at how individual characteristics affect incentives to learn and look at extra technological aspects that affect IoT uptake. Subsequent research could improve the assessment and give more insight into the value of IoT by including mediating factors between personal characteristics and technological attributes. The theoretical framework could also be used with cutting-edge technology like metaverse systems and artificial intelligence.

## Abbreviations

ANN: artificial neural network
AVE: average-variance extracted
INT: intention to use Internet of Things
IoT: Internet of Things
IPMA: importance-performance map analysis
LMT: learning motivation
PE: perceived ease of use
PLS-SEM: partial least squares–structural equation modeling
PU: perceived usefulness
RMSE: root mean square of error
SEM: structural equation modeling
TAM: technology acceptance model
TIN: technology innovativeness
TOP: technology optimism
